# Electromyographic and Kinematic Analysis of the Upper Limb During Drinking and Eating Using a Wearable Device Prototype

**DOI:** 10.3390/s25175227

**Published:** 2025-08-22

**Authors:** Patrícia Santos, Filipa Marquês, Carla Quintão, Cláudia Quaresma

**Affiliations:** 1 Laboratory for Instrumentation, Biomedical Engineering and Radiation Physics (LIBPhys), NOVA School of Science and Technology, NOVA University of Lisbon, 2829-516 Caparica, Portugal; par.santos@campus.fct.unl.pt (P.S.); cmquintao@fct.unl.pt (C.Q.); 2 Associated Laboratory in Translation and Innovation Towards Global Health (REAL), NOVA School of Science and Technology, NOVA University of Lisbon, 2829-516 Caparica, Portugal; 3 Physics Department, NOVA School of Science and Technology, NOVA University of Lisbon, 2829-516 Caparica, Portugal; fmv.marques@campus.fct.unl.pt; 4 Health Department, Superior School of Health, Polytechnic Institute of Beja, 7800-111 Beja, Portugal

**Keywords:** wearable sensors, electromyography, inertial measurement units, upper limb, activities of daily living, biomechanics

## Abstract

The assessment of upper limb (UL) movement patterns plays a critical role in the rehabilitation of individuals with motor impairments resulting from neuromotor disorders, which significantly affect essential activities of daily living (ADLs) such as drinking and eating. However, conventional clinical evaluation methods often lack objective and quantitative insights into the biomechanics of movement. To enable accurate identification of pathological patterns, it is first necessary to establish normative biomechanical and electrophysiological benchmarks in healthy individuals. In this study, a previously developed, low-cost, wearable, and portable prototype device was employed to objectively assess UL movement. The system, specifically designed for clinical applicability, integrates surface electromyography (EMG) sensors and an inertial measurement unit (IMU) to capture muscle activity and kinematic data, respectively. Thirty healthy participants were recruited to perform standardized drinking and eating tasks. The analysis focused on characterizing muscle activation patterns and joint range of motion during different task phases. Results revealed consistent variations in muscle contraction and joint kinematics, allowing the identification of distinct activation profiles for key shoulder muscles. The findings contribute to the establishment of a normative dataset that can serve as a reference for the assessment of clinical populations. Such data are essential for informing rehabilitation strategies and developing predictive models of UL function during ADLs in individuals with neuromotor disorders.

## 1. Introduction

Upper limb (UL) performance in ADLs can be significantly compromised by motor functional limitations, particularly those caused by neurological disorders [1,2,3]. Since motor dysfunction is a common complication of stroke, and the most frequent deficit is hemiparesis of the contralateral UL, with more than 80% of stroke patients experiencing this condition in the acute phase [4], their ability to perform ADLs is impaired. It is estimated that 37–55% of subjects with stroke have deficits in the performance of these activities [5], related to the omission of small actions and changes in the sequence and the quality, as demonstrated in studies related to the preparation of meals [6] and hygiene [7].

One of the most common issues associated with UL recovery in stroke patients is the development of a compensatory movement pattern. These are based on altered movement patterns involving the contraction of muscles that would not normally be expected to activate during a specific movement, and they arise due to reduced strength, abnormal muscle tone, pain, and other contributing factors [8,9]. These compensatory movements may be detrimental to the long-term functional outcome of the affected arm, as they often result in inefficient patterns that limit progress and hinder recovery of the paretic limb [10,11]. This underscores the importance of monitoring compensatory movements to optimize rehabilitation strategies for stroke survivors [12].

An objective and precise analysis of UL neuromotor patterns using quantitative data is essential to prevent such issues [13], as it ensures greater accuracy than conventional clinical methods, which often rely on subjective scales and high-variability tools like manual goniometers. These outcome measures are reliable and sensitive for measuring gross changes in functional performance, but have less sensitivity to smaller and more specific changes. Furthermore, despite the extensive experience in using these observer-initiated measures by clinicians, the subjectivity of these tests cannot be denied [14].

The rehabilitation of motor functions is fundamental to restoring quality of life following a stroke. However, advancing effective and innovative strategies beyond conventional rehabilitation protocols remains both essential and challenging [15].

Sensor-based technologies have emerged as valuable tools for the quantitative analysis of movement patterns associated with ADLs [16], providing high-resolution data that surpasses the precision and objectivity of traditional clinical assessments based solely on observational scales [17]. The integration of biomechanical and electrophysiological signals enables the identification of deviations in motor execution across different phases of functional tasks. By analyzing these multimodal data, it becomes possible to distinguish between typical and atypical movement patterns, thereby facilitating the early detection of neuromotor dysfunction and supporting personalized rehabilitation strategies [17].

Prevention of compensatory movement is considered best practice for early detection and retraining of appropriate motor patterns. These approaches are grounded in one of the most influential theories in neurorehabilitation: the Motor Development Theory, commonly operationalized through Neurodevelopmental Treatment (NDT), also known as the Bobath Concept, which emphasizes the natural developmental sequence of motor control from proximal to distal regions [18]. According to this theory, achieving stability in the proximal upper limb (i.e., shoulder) is a prerequisite for effective distal function. Therapists initially target proximal segments to inhibit abnormal compensatory strategies and facilitate more normalized motor patterns. Once proximal control is achieved, fine manual skills can be trained independently, a progression widely used in stroke rehabilitation to promote refined, dissociated distal movements [18].

Prescribing restorative interventions is essential to understand movement patterns and underlying motor strategies [19]. To this end, it is first necessary to gain knowledge about the neuromotor profile dynamics in healthy individuals, particularly regarding muscle activation patterns and joint angles during activities of daily living (ADLs), which are most frequently affected after stroke [6,7]. This prior understanding of the neuromuscular activation dynamics—specifically, the movements performed in each phase of the ADLs and the corresponding agonist muscles—will be essential for future comparisons with individuals with neuromotor pathologies. It will support the identification of compensatory movements, alterations in the sequence and quality of movements, and the omission of specific subtasks within these activities [5].

Although there are studies that have analyzed the motor pattern of the UL during ADLs, they focus solely on the use of EMG [20] or only on the use of IMU [18], or optoelectronic motion capture camera systems [14,19,21,22,23,24,25,26,27], or consider only simple functional tasks rather than in all ADLs performance [28,29,30]. Few studies have focused on the simultaneous analysis of kinematic and electrophysiological parameters in the upper limb [31], as most do not integrate EMG and IMU signals within a single device—hindering precise signal synchronization—or present portability constraints that limit their applicability in daily life contexts, since they are mainly used in laboratory environments [32].

Additionally, one of the most recent systematic reviews on the use of wearable technologies to monitor individuals with neurological impairments during ADLs [33] further supports the observations. Among the studies referenced in this review, none reported the use of both EMG and IMU to monitor the neuromotor pattern dynamics at the proximal region of the upper limb (i.e., the arm) during the execution of ADLs.

Most of the wearable technologies identified in the included studies consisted of the isolated use of accelerometers, IMU, or EMG sensors, primarily positioned on distal segments of the upper limb, such as the forearm, wrist, and fingers. Only two studies [34,35] addressed the proximal upper limb, and even then, solely by IMUs. These studies focused on variables related to wheelchair mobility ADLs, specifically during wheelchair propulsion, which involves different biomechanical and functional requirements compared to eating and drinking. Moreover, the analyses were limited to variables such as humeral elevation amplitude, acceleration, and angular velocity.

Another valuable contribution is provided by a recent article [36] presenting an EMG dataset for the classification of ADLs. However, it focuses solely on the EMG activity of the hand segment during isolated tasks, rather than during the performance of essential ADLs that are critical for human life and autonomy, such as eating and drinking.

As a result, there is limited scientific evidence in this field and a lack of studies using integrated EMG and IMU devices to analyze movement patterns during the execution of ADLs that are portable and user-friendly, and therefore suitable for application in various contexts [37,38]. The integration of IMU and EMG sensors for UL analysis provides significant advantages in the field of rehabilitation, enabling the correlation between kinematic data and neuromuscular activity [39].

EMG measures the electrical activity associated with muscle activation, where increased recruitment of motor units results in higher signal amplitudes and stronger muscle contractions [40]. However, EMG also presents limitations, as factors such as fatigue [41] and movement speed [42] may influence and increase muscle activation amplitude. EMG provides insights into muscle contraction and relaxation during movement, enabling a better understanding of contraction patterns and the identification of potential muscle compensations during ADLs [43].

This information is supported by IMU data, as movement in each segment only occurs when the agonist and synergist muscles responsible for that motion are activated. IMU sensors combine an accelerometer, gyroscope, and magnetometer in a single module, enabling the measurement of acceleration, angular velocity, and spatial orientation. Their internal processing also allows the calculation of Euler angles (roll, pitch, and yaw), providing a comprehensive analysis of movement dynamics and spatial positioning [44]. IMU sensors are commonly used to assess joint-related variables in limb segments such as the arm, forearm, and hand, offering data on position, velocity, and range of motion with up to nine degrees of freedom [39].

The analysis of the neuromotor pattern through a prototype of a portable, low-cost device integrating EMG and IMU, allowing the evaluation in any context, is essential for the rehabilitation process. Only by this integrated data is it possible to know the neuromotor pattern, analyzing the activation amplitude of a specific muscle group, synchronized with the degrees of movement performed by the respective segment.

This study aims to explore the neuromotor dynamics of the arm segment in both upper limbs—specifically muscle activation amplitude and triaxial joint motion—using a portable, low-cost, and user-friendly device developed with integrated EMG and IMU sensors. The analysis is conducted during two ADLs that are highly susceptible to compensatory movements, namely the activities of eating and drinking [45].

To this end, the activity of the primary shoulder muscles was analyzed [46]: Pectoralis Major (PM), Anterior Deltoid (AD), Middle Deltoid (MD), Posterior Deltoid (PD), Upper Trapezius (UT), and Lower Trapezius (LT), along with triaxial shoulder movements of the arm segment, including Flexion (F), Extension (E), Medial Rotation (MR), Lateral Rotation (LR), Abduction (ABD), and Adduction (ADD).

The central research question is whether this device can effectively characterize the bilateral neuromotor patterns of the upper limbs during these ADLs in healthy individuals. Additionally, the study aims to contribute to the development of normative references for upper limb neuromotor patterns, providing a baseline for future investigations in stroke populations and supporting the clinical understanding of shoulder muscle function during ADLs.

## 2. Materials and Methods

### 2.1. Wearable Device Prototype and Interface

**Figure 1:**
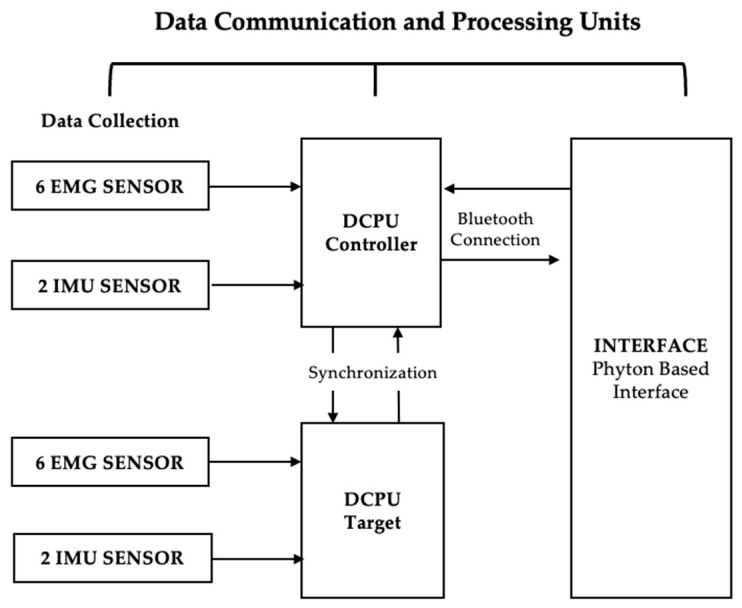
Block diagram illustrating the data communication and processing architecture, where the Controller and Target DCPUs receive input from six EMG sensors and two IMU sensors each, with the Controller handling synchronization and Bluetooth transmission to a Python-based interface.

This study used a prototype wearable device consisting of a portable, low-cost data acquisition system for kinematic and electrophysiological analysis (Figure 1). Hardware and a software interface were developed to acquire, communicate, and visualize EMG and IMU data for neuromotor pattern analysis. The device was created using a co-creation methodology involving researchers, engineers, and healthcare professionals.

The electronic circuits, designed by WallySci (Neuriot Technologies LLP, Faridabad, India), comprise two Data Communication and Processing Units (DCPUs), each with an ESP32-WROOM-32D microcontroller powered by a battery and integrated on a board with 10 GPIO pins, six 12-bit analog inputs, and two 8-bit analog outputs. Each DCPU connects via I2C to six EMG sensors and one 9DoF IMU sensor, transmitting data wirelessly to the graphical interface via Bluetooth. All these components were designed by the company WallySci (Neuriot Technologies LLP, Faridabad, India).

Two microcontrollers (Controller and Target) were programmed in the Arduino IDE 2 and connected via Bluetooth to each other and the graphical interface. When the interface sends command 1, the Controller triggers a sync pulse from its syncPin (output) to the Target´s syncPin (input), aligning data acquisition. EMG signals were sampled at 1000 Hz (analog pins 36, 39, 32, 34, 35, 33) and IMU data at 100 Hz. The I2C bus was initialized with Wire.begin() and the IMU with imu.begin(). Both microcontrollers ran a continuous loop to monitor data availability. When detected, imu.dataAvailable() retrieved orientation quaternions, later converted to Euler angles (yaw, pitch, and roll).

**Figure 2:**
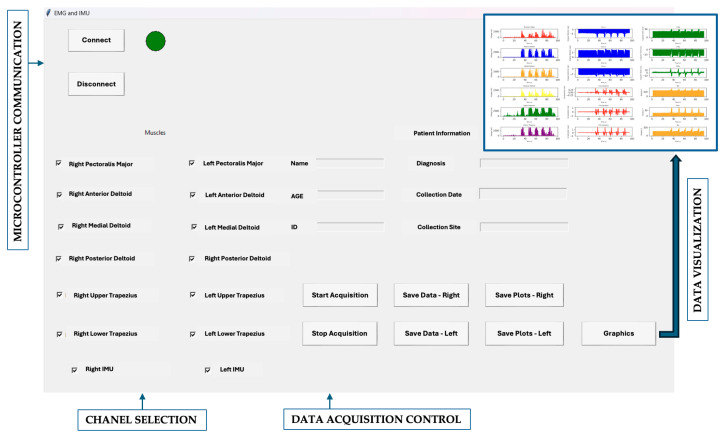
Interface developed for microcontroller communication, enabling data acquisition control, channel selection, and graphical visualization of EMG and IMU signals.

An interface based on Python 3.11.10 (Figure 2) was developed to enable seamless interaction between the portable device and a computer, focusing on real-time data visualization. It includes a button to connect to the microcontroller with feedback confirmation, controls to start/stop data acquisition, and customizable channel selection. Users can display data from individual sensors for each limb, enter patient information, and view results as graphs showing muscular activity from six muscles and rotational angles (roll, pitch, yaw), color-coded and with units indicated. Designed for usability, the tool improves data accessibility and interpretability, supporting more effective analysis and decision-making.

### 2.2. Subject Recruitment

An initial sample of 32 healthy participants was recruited; 2 were excluded due to Bluetooth connectivity issues between the device and the computer during data recording, resulting in a final sample of 30 individuals (17 females, 13 males; all right-handed; mean age 35.2 ± 10.2 years; mean height 172 ± 5 cm). Inclusion criteria comprised adults aged ≥18 years with no self-reported history of musculoskeletal, neurological, or cognitive disorders that could affect upper limb motor control. All participants provided written informed consent prior to participation, and anonymity was ensured. The study was conducted in accordance with the Declaration of Helsinki and was approved by the Ethics Board of NOVA’s Faculty of Science and Technology (CE_FCT_002_2022).

### 2.3. Experimental Setup and Protocol

**Figure 3:**
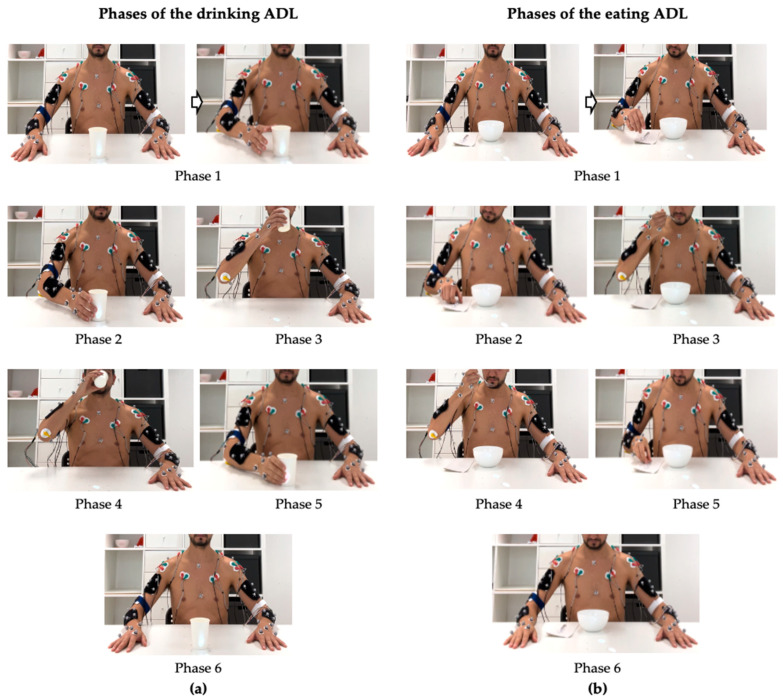
ADLs phases abbreviation: Phase 1, starting position to reaching; Phase 2, grasping; Phase 3, transporting to the mouth; Phase 4, introduced in the mouth; Phase 5, return to the table; Phase 6, return to initial position. (**a**) Graphical representation of the six phases of the drinking ADL. (**b**) Graphical representation of the six phases of the eating ADL.

A protocol was adapted from previous studies [21,31,47], and the participants were seated in the same position as in those studies. For the drinking ADL, a plastic cup was placed 20 cm from the edge of the table and aligned with the subject’s midline. For the eating ADL, a soup bowl was positioned 3 cm from the edge of the table, also aligned with the subject’s midline, and a spoon was placed next to the bowl on the subject’s dominant side. The activity was first performed at a comfortable speed [23]. Five attempts were repeated with a 5 s rest to minimize fatigue. One camera with a resolution of 30 frames/s (coronal plane) was used to establish the six phases of the ADL, from Phase 1 to Phase 6 (Figure 3).

These phases consist of dividing the activities into smaller tasks (Figure 3): Phase 1, “Starting position to reaching,” corresponds to the interval from the moment the hand is lifted from the table to the point at which the upper limb positions the hand near the object (cup or spoon), initiating the grasping process, while preparing for interaction but before any actual contact is established; Phase 2, “Grasping,” encompasses the interval from the moment the hand reaches the position of approach, just before contact with the object, until the fingers effectively make contact, that is, the act of grasping, and the object is securely held; Phase 3, “Transporting to the mouth,” begins immediately after the object has been securely grasped and lifted from the table, and continues until the moment just before the object makes contact with the mouth; Phase 4, “Introduction in the mouth,” begins when the object (spoon or cup) makes initial contact with the mouth and ends when it has fulfilled its function, such as delivering food or liquid and makes its final contact with the mouth before being withdrawn; Phase 5, “Return to the table”, occurs from the moment the object leaves the mouth until it is placed back on the table; and Phase 6, “Return to starting position,” begins when the hand releases contact with the object and returns to its initial grasping position, marking the moment when the flexor muscles of the hand begin to relax and the finger extensors are activated to release the object with precision and withdraw the hand, allowing the upper limb to return to its original starting position. Subsequently, an additional phase was considered in the activity cycle: Phase 7, “Rest”, which reflects the decrease in neuromotor activity after the upper limb returns to its starting position. It is also important to highlight that the division of these ADLs (drinking and eating) was based on previous studies [15,21], as well as an adaptation of the grasping phases established by Kapandji [48], in order to allow a more precise analysis of the neuromotor pattern dynamics of the proximal region of the upper limb (arm) during each phase, according to the specific demands of each activity.

**Figure 4:**
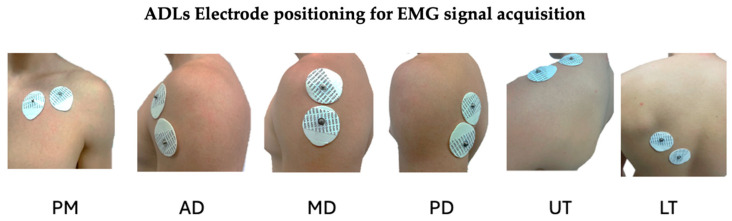
Electrode placement for EMG signal acquisition using two surface electrodes in a bipolar configuration, enabling differential signal detection and noise minimization, applied to the following muscles: Pectoralis Major (PM), Anterior Deltoid (AD), Middle Deltoid (MD), Posterior Deltoid (PD), Upper Trapezius (UT), and Lower Trapezius (LT).

EMG and IMU data were acquired using the device prototype, which was connected via Bluetooth to the software running on a computer. The EMG sensors were placed in muscles, both ULs, using the bipolar method, defined by SENIAM, and were placed in the same positions as described in Figure 4. The 2 IMU, placed above the lateral epicondyle, and the reference electrode on the olecranon [49].

### 2.4. Data Analysis

**Figure 5:**
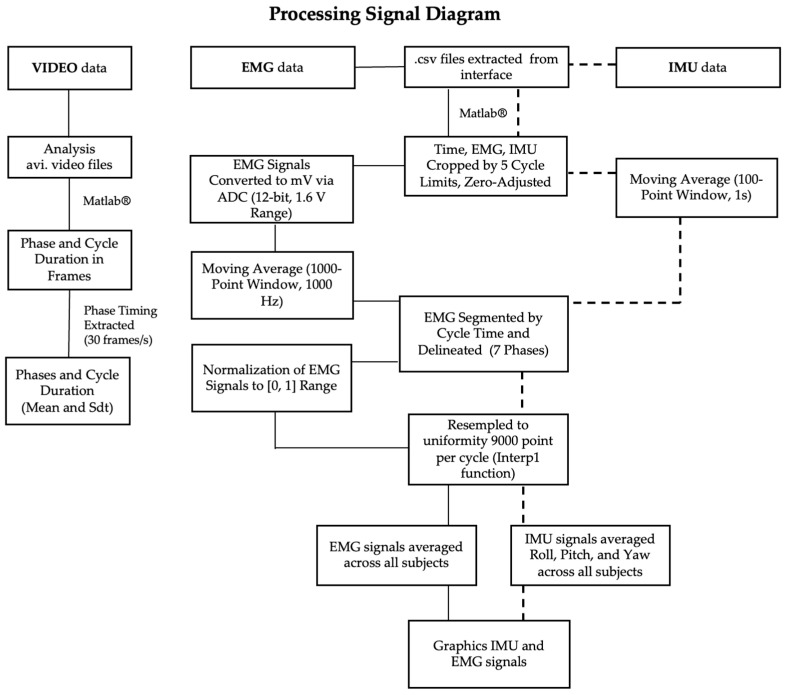
Block diagram illustrating the processing of data from video, EMG, and IMU.

To define ADL phases, camera videos (.avi) were processed in Matlab R2025a^®^ frame-by-frame to record the start and end times of each phase in an Excel file, using frame rate to calculate duration, mean, and SD. EMG data (.csv) were imported, retaining time and channel-specific signals. Five cycles per subject were trimmed to subject-specific limits, time-normalized to zero, and converted from bits to mV (12-bit resolution, 1.6 V range). Signals were smoothed with a 1000-sample moving average (1 s at 1000 Hz), segmented according to phase times from DurationCycle.xlsx, and pauses isolated. Cycles were resampled to 9000 points using interp1, averaged, normalized (0–1), and then averaged across subjects. IMU data (.csv) were trimmed to subject-specific limits, time-reset to zero, and smoothed with a 100-point moving average (1 s). Signals (Roll, Pitch, Yaw) were segmented into predefined movement phases, aligned to start at zero, resampled to 9000 points, and averaged across subjects. A summary of the processing steps and algorithms employed is presented in the diagram below (Figure 5).

## 3. Results

The results are structured based on the ADLs examined, beginning with the presentation of findings for the drinking and eating tasks.

### 3.1. ADL of Drinking

Regarding the definition of phases comprising the drinking activity cycles, the phases with the longest duration are Phase 4 (21.7% ± 5.9), Phase 6 (17.9% ± 7.4), and Phase 1 (17.2% ± 3.5), while the shortest is Phase 2 (4.4% ± 4.0) (Table 1).

**Figure 6:**
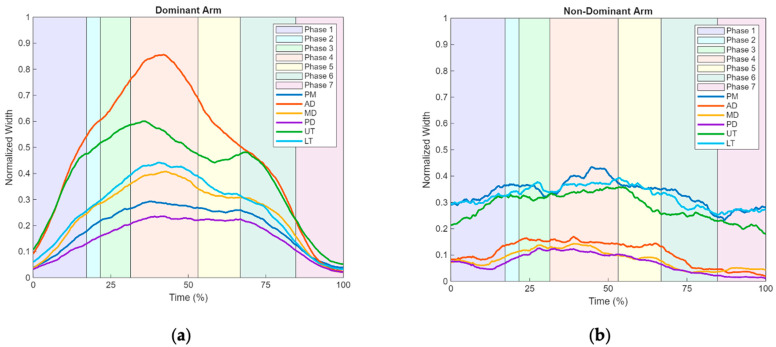
Graphical representation of the activation amplitude pattern of the six analyzed muscles (PM, AD, MD, PD, UT, LT) across the different phases of the drinking ADL in upper limb: (**a**) dominant arm. (**b**) Non-dominant arm.

Regarding the EMG results, Figure 6a,b present the mean muscle activation amplitude of the AD, PD, MD, PM, UT, and LT during the drinking activity for both ULs.

In all muscles of the dominant arm, as well as in the PM, AD, and LT muscles of the non-dominant arm, a progressive increase in activation amplitude was observed, reaching peak amplitude in Phase 4. These results are presented in Table 2, showing the maximum activation peaks of the muscles in the dominant arm (AD 0.7 mV at 42.0%, UT 0.6 mV at 36.1%, LT 0.4 mV at 40.7%, MD 0.4 mV at 44.0%, PM 0.3 mV at 38.3%, and PD 0.2 mV at 42.6%) and in the non-dominant arm (PM 0.4 mV at 44.9%, AD 0.2 mV at 39.1%, and MD 0.1 mV at 40.2%). The peak activation of the PD, LT, and UT muscles in the non-dominant arm occurred, respectively, in Phase 2 for the PD (0.1 mV at 28.0%) and in Phase 5 for both the LT (0.4 mV at 53.3%) and UT (0.4 mV at 54.8%).

A subsequent decline in activation is observed in all muscles of the dominant arm (Figure 6a) up to the end of Phase 5, with an inflection point at the beginning of Phase 6 in all muscles except the AD, which shows a more continuous decline until Phase 7. In the muscles of the non-dominant arm (Figure 6b), no marked decline in activation is observed following the peak amplitude, as seen in the dominant arm. However, a gradual decrease in activation amplitude is evident in the PD, MD, and AD muscles up to Phase 7, while the PM, UT, and LT muscles show a slight inflection point in Phase 6, without a pronounced reduction in activation throughout Phases 6 and 7.

**Figure 7:**
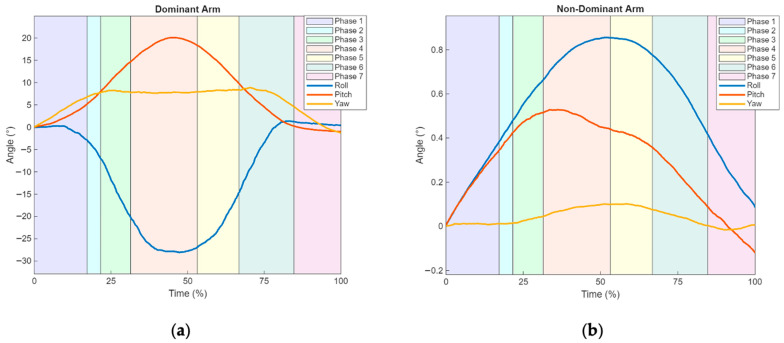
Graphical representation of mean arm joint range of movement in the six possible directions across the different phases of the drinking ADL for the upper limbs: (**a**) Dominant Arm—Flexion (F, −Yaw), Extension (E, +Yaw), Adduction (ADD, −Roll), Abduction (ABD, +Roll), Medial Rotation (MR, −Pitch), and Lateral Rotation (LR, +Pitch); (**b**) Non-dominant Arm—Flexion (F, −Yaw), Extension (E, +Yaw), Adduction (ADD, +Roll), Abduction (ABD, −Roll), Medial Rotation (MR, +Pitch), and Lateral Rotation (LR, −Pitch).

The data obtained from the IMU sensors provide information on the joint angle amplitudes of both the dominant and non-dominant arms, as shown in Figure 7a,b, and Table 3.

Regarding the average joint range of motion in the dominant arm (Figure 7a and Table 3), during Phase 4 (corresponding to 31.4% and 53.2% of the task cycle as shown in Table 1), there is an increase in ADD (peak maximum of −29.0° at 47.7%), LR (peak maximum of −21.0° at 45.5%), and E (peak maximum of 8.9° at 70.3%).

Although the peak amplitude of E occurs in Phase 6, a pronounced increase is observed up to Phase 3, followed by a stabilization period until Phase 6, when it reaches its maximum, after which a reversal toward F begins (peak maximum of −0.2° at 90.6% of the cycle during Phase 7).

Similarly, after the peak in ADD, a reverse movement toward ABD is observed, reaching its maximum of 1.4° at 83.0% in Phase 7. The same occurs after the LR peak, with a reverse movement toward MR, culminating in a peak of −1.0° at 100.0%.

In the non-dominant upper limb (Figure 7b and Table 3), an increase in joint range of motion is observed, with all movements reaching their peak values in ADD (0,9° at 52.5%) and E (0.1° at 58.5%) during Phase 5 (53.2° to 66.8% of Table 1), and MR (0.5° at 36.2%) during Phase 4 (31.4% and 53.2% of Table 1). Following these peaks, reverse movements are initiated, ABD (peak of 1.0° at 100.0%) and LR (peak of −0.1° at 100.0%). After the peak of E (0.10° at 58.5%), the reverse movement of F begins, reaching −0.2° at 84.7% by the end.

### 3.2. ADL of Eating

With respect to the characterization of the phases constituting the ADL of eating, the phases exhibiting the longest durations are Phase 3 (23.0% ± 3.0), Phase 6 (16.8% ± 3.9), and Phase 1 (15.9% ± 2.5), while the shortest is Phase 2 (5.2% ± 1.7) (Table 4).

**Figure 8:**
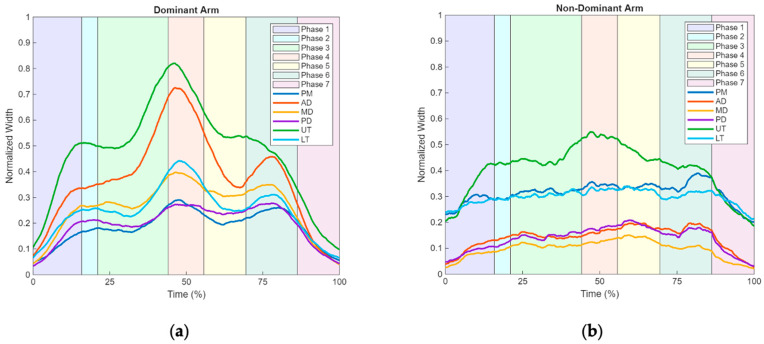
Graphical representation of the activation amplitude pattern of the six analyzed muscles (PM, AD, MD, PD, UT, LT) across the different phases of the eating ADL in upper limb: (**a**) dominant arm. (**b**) Non-dominant arm.

The EMG results in Figure 8a,b display the mean muscle activation amplitudes across all assessed muscles during the drinking task for both upper limbs. In all muscles of the dominant arm (Figure 8a), a progressive increase in activation amplitude was observed, reaching peak amplitude in Phase 4, (44.1% and 55.7%, Table 4) (UT 0.8 mV at 46.1%; AD 0.7 mV at 46.3%; LT 0.4 mV at 47.9%; MD 0.3 mV at 46.7%; PM 0.3 mV at 47.8%), as shown in Table 5. The exception is PD (0.3 mV at 78.0%), reaching peak amplitude in Phase 5 (Table 5). A subsequent decline in activation is observed in all muscles of the dominant arm (Figure 8a) up to the midpoint of Phase 5, followed by an inflection point extending into Phase 6 in all muscles except the Upper Trapezius (UT), which does not exhibit a pronounced inflection. Thereafter, all muscles show a continuous decrease in activation until the end of Phase 7.

In the muscles of the non-dominant arm (Figure 8b), no marked decline in activation is observed following the peak amplitude, as seen in the dominant arm. In muscles of the non-dominant arm (Figure 8b), a progressive increase in activation amplitude is observed, reaching the peaks amplitude in Phase 5 (between 55.7% and 69.5%, Table 1) (LT 0.3 mV at 58.1%, PD 0.2 mV at 60.0%, and AD 0.2 mV at 79.5%), Phase 4 (between 44.1% and 55.3% Table 1) (UT 0.6 mV up to 47.0% and MD 0.2 mV up to 52.2%), and step 6 (PM 0.4 mV up to 82.0%). In muscles of the non-dominant arm (Figure 8b), a progressive increase in activation amplitude is observed, reaching peak amplitudes in Phase 5 (between 55.7% and 69.5%, Table 1) (LT 0.3 mV at 58.1%, PD 0.21 mV at 60.0%, and AD 0.2 mV at 79.5%), Phase 4 (between 44.1% and 55.7% Table 1) (UT 0.6 mV at 47.0% and MD 0.2 mV at 52.2%), and Phase 6 (PM 0.4 mV at 82.0%). After reaching the maximum amplitude peaks, a gradual decrease in activation amplitude is observed.

**Figure 9:**
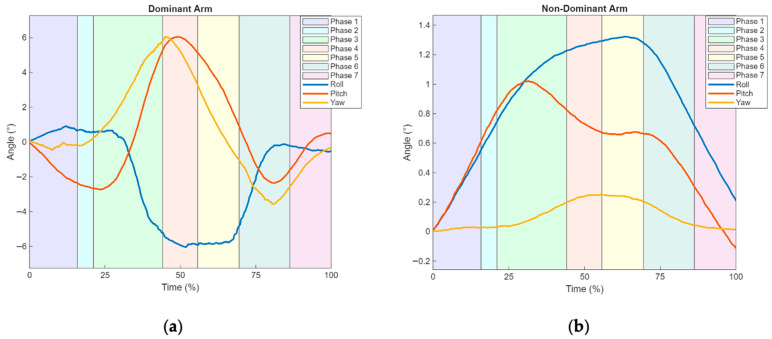
Graphical representation of mean arm joint range of movement in the six possible directions across the different phases of the eating ADL for the upper limbs: (**a**) Dominant Arm—Flexion (F, −Yaw), Extension (E, +Yaw), Adduction (ADD, −Roll), Abduction (ABD, +Roll), Medial Rotation (MR, −Pitch), and Lateral Rotation (LR, +Pitch); (**b**) Non-dominant Arm—Flexion (F, −Yaw), Extension (E, +Yaw), Adduction (ADD, +Roll), Abduction (ABD, −Roll), Medial Rotation (MR, +Pitch), and Lateral Rotation (LR, −Pitch).

Data obtained from the IMU sensors provide information on joint angle amplitudes of both the dominant and non-dominant arms, as presented in Figure 9a,b, and summarized in Table 6. In the dominant arm (Figure 9a and Table 6), during Phase 4 (corresponding to 44.1% and 55.7% of the task cycle, as shown in Table 4), there is an increase in E (maximum peak of 6.1° to 47.7%), LR (maximum peak of 6.1° to 49.2%), and ADD (maximum peak of −6.0° 51.7%). Although the peak amplitude of the LR occurs in Phase 4, there is an inflection point earlier in Phase 3, at which the maximum peak of the MR joint amplitude is reached (−2.7° to 23.9%). The same occurs with the ABD movement (12.1° to 23.8%) in Phase 1.

Inflection peaks are also observed during Phase 6 of the activity cycle. After reaching its maximum peak in Phase 4, the Lateral Rotation (LR) transitions into Medial Rotation (MR), which continues until the end of Phase 6. At this point, an inflection occurs, marking the return to LR, which persists until the final phase of the activity cycle. E follows a similar pattern, demonstrating analogous variations in the joint angle amplitude throughout the movement cycle.

The same is observed for ADD, which presents an inflection point at the beginning of Phase 6, marking the initiation of ABD. This Abduction movement continues and stabilizes by the end of the same phase, remaining constant until the completion of the eating activity cycle. In ADD, an inflection point is also observed at the beginning of Phase 6, marking the initiation of ABD. This Abduction movement continues and stabilizes by the end of the same phase, remaining consistent until the completion of the eating activity cycle.

In the non-dominant upper limb (Figure 9b and Table 6), an increase in the range of joint motion was observed, with all movements reaching their maximum values in ADD (1.3° to 63.6%) during Phase 5 (between 55.7% and 69.5%), RM (1.0° to 31.5%) during Phase 3 (between 21.1% and 44.1%), and E (0.2° to 55.4%) during Phase 4 (between 21.1% and 44.1%). Following these peaks, reverse movements of ABD, LR, and F are initiated and continue until the end of the activity cycle.

## 4. Discussion

The patterns of muscle activation amplitude and joint range of motion amplitude are two key parameters that enable a more accurate characterization of the neuromotor profile of the upper limb. By IMU sensors integrated into wearable devices alongside EMG sensors, it becomes possible to synchronously characterize human movement during ADLs [38].

The results of this study offer a refined understanding of bilateral upper limb synergies, particularly regarding the relationship between arm segment movements and the activation patterns of agonist and antagonist muscle groups during the execution of two essential ADLs: eating and drinking. These tasks are fundamental for individual autonomy and well-being and are especially susceptible to compensatory motor strategies in individuals with neuromotor disorders.

A multidimensional analysis strategy was employed, integrating biomechanical and electrophysiological data collected through synchronized IMU and EMG. This dual-sensor approach enabled a comprehensive evaluation of bilateral upper limb motor patterns during the performance of the selected ADLs. Although partial comparisons were made with findings reported in prior studies [27,31,38,47], it is important to note that previous research has typically relied on isolated sensor modalities and predominantly unimanual analyses, limiting direct comparisons. To the best of our knowledge, no previous study has applied an integrated IMU and EMG system to analyze the bilateral performance of these specific ADLs.

A consistent neuromotor pattern emerged in the dominant limb across both tasks. Specifically, EMG and IMU signals displayed a similar temporal profile: an initial phase of increasing amplitude culminating around Phase 4, followed by a progressive decrease through the later stages of the movement and into the final resting phase (Phase 7). This temporal alignment between EMG amplitude and joint range of motion suggests a clear synergy between muscle activation and segmental displacement during functional movement execution.

Such findings reinforce fundamental neuromechanical principles, wherein upper limb motion requires the orchestrated contraction of agonist muscles to produce movement about the joint. This contraction is typically reflected in elevated EMG signal amplitude and corresponds with increased joint excursion as recorded by the IMU system. For example, activation of the AD and UT during arm Flexion or Abduction aligns closely with angular displacement in the sagittal and frontal planes. The decline in both EMG and kinematic signals observed in later phases of the task reflects expected deceleration and stabilization as the limb completes the intended motion.

Overall, the simultaneous analysis of muscle recruitment and joint motion in healthy participants contributes to a better understanding of upper limb neuromotor patterns during key ADLs. This data holds significant potential of knowledge for future studies aiming to establish normative patterns and for future clinical applications, particularly for the early detection of abnormal motor strategies and the development of personalized rehabilitation protocols in populations affected by neuromotor impairments, such as stroke survivors.

Regarding the dominant arm in the drinking activity (Table 3), and in comparison with other studies that used IMU to analyze shoulder joint range of motion [25,26], the results obtained from healthy subjects in the control group show higher values than those observed in the present study, specifically in terms of the differences between the peak amplitudes of arm movements (F/E, ABD/ADD, and LR/RM). This discrepancy may be explained by differences in the protocols used to perform the drinking task. However, the differences between the peak amplitudes of ABD/ADD are similar to those reported in previous studies [15,24]. From a comparative perspective with other studies [47] and now focusing on the muscle activation amplitude patterns obtained through EMG (Figure 6a), similarities are observed both in the activation patterns across the six analyzed muscles (PM, AD, MD, PD, UT, LT) and the timing of peak contractions. These consistencies further support the findings obtained in the present study.

In addition to the limited comparability with previous studies, primarily due to differences in experimental protocols and the specific kinematic variables analyzed, the present study offers relevant insights into the neuromotor characterization of the drinking activity. By examining the contraction patterns of both the dominant and non-dominant upper limbs during this task, it was observed that, although the non-dominant limb remained passively supported on the table without any assigned functional role, a degree of synergistic muscular activation was still present. Both limbs exhibited similar temporal activation patterns, with peak muscle activation amplitudes occurring in Phase 4 across all muscles, except for the PD, which reached its peak in Phase 3.

When comparing these muscle activation patterns with the corresponding joint range of motion, it becomes evident that both signals reach their maximum values during Phase 4. In the subsequent phases, particularly during the deceleration of the movement, an inflection in the amplitude of the MD and UT signals was observed. This finding is consistent with the functional roles of these muscles, as the UT is primarily responsible for shoulder elevation, and the MD is an agonist in arm ABD. During Phase 6, which involves the return of the upper limb to the initial resting position, these muscles are again recruited due to the combined actions of F and ABD required to control the movement, thereby justifying the observed changes in signal amplitude.

In contrast, the neuromotor pattern recorded during the eating activity reveals a more complex and undulating profile, differing considerably from that observed in the drinking task. The data show the presence of two inflection points, one occurring prior to and another following the peak of muscle activation. A similar dual-phase pattern was also evident in the joint range of motion signals, suggesting the involvement of a more sophisticated motor strategy to execute the task. This complexity may be attributed to the multiple phases of the activity, which include grasping the utensil, guiding it to the mouth, and subsequently returning it to the support surface.

These observations are consistent with previous studies that analyzed muscle activation during eating, which reported a preliminary phase of moderate activation followed by a progressive increase that culminates in a peak at the central phase of the task. This is succeeded by a clear reduction in activation amplitude, particularly noticeable in the AD, UT, and MD. The first inflection point is associated with the initiation of the functional movement, such as grasping and lifting the spoon, while the second reflects the repositioning of the utensil, tasks that require fine motor coordination and varying muscular engagement across different muscle groups.

Overall, these results provide a refined understanding of the bilateral neuromotor synergies involved in two fundamental ADLs. The use of synchronized EMG and IMU data enables the precise characterization of the interaction between muscle activity and joint kinematics. This contributes not only to the identification of normative patterns in healthy individuals but also lays the groundwork for future comparative analyses in clinical populations, where deviations from these patterns may serve as diagnostic indicators or targets for personalized rehabilitation strategies.

Regarding the synergies established between the dominant (Figure 6a) and non-dominant (Figure 6b) arm during this activity, it is observed that, although the non-dominant limb exhibits lower overall muscle activation, it subtly mirrors the same activation pattern. This finding supports the results also observed during the drinking ADL, suggesting the presence of associated bilateral responses between both arms. The present study identified a consistent neuromotor pattern in arm function, characterized by seven distinct phases of synchronized muscle activation and joint kinematics. This pattern was observed in both dominant and non-dominant arms, even in the absence of active participation from the non-dominant side, suggesting the existence of inherent bilateral motor synergies during the execution of ADLs. Furthermore, the identification of inflection points in both electromyographic and kinematic signals reinforces the interdependence between muscle activity and joint movement in functional motor tasks.

## 5. Conclusions

These findings underscore the added value of integrating inertial measurement units (IMUs) and surface electromyography (EMG) within a single, portable, and user-friendly system for comprehensive human movement analysis. The use of this integrated system enabled the simultaneous acquisition and synchronized analysis of kinematic and electrophysiological data, offering a detailed characterization of upper limb motor strategies during two essential ADLs (eating and drinking).

By combining EMG and IMU data, this study provides novel insights into the functional relevance of neuromotor patterns, particularly in the context of neurorehabilitation. The ability to detect subtle bilateral responses in tasks typically considered unilateral opens new perspectives for the development of rehabilitation protocols that enhance functional synergies, improve motor coordination, and promote autonomy in individuals with neuromuscular impairments.

Future studies should aim to expand the sample size, investigate a broader range of ADLs, and include participants across different age groups and clinical populations, particularly those affected by neurological disorders. A deeper understanding of neuromotor strategies, reflected in phase-specific patterns of muscle activation, joint range of motion, and signal inflection points, has the potential to inform the design of more effective, individualized rehabilitation interventions grounded in objective biomechanical and electrophysiological evidence.

## Figures and Tables

**Table 1:** Mean normalized time interval(s) and mean phase duration (%) of the drinking ADL phase.

Phases	Interval of Mean Time (%)	Mean Duration ± SDT (%)
1	[0, 17.2]	17.2 ± 3.5
2	[17.2, 21.6]	4.4 ± 4.0
3	[21.6, 31.4]	9.8 ± 4.8
4	[31.4, 53.2]	21.7 ± 5.9
5	[53.2, 66.8]	13.6 ± 2.6
6	[66.8, 84.7]	17.1 ± 7.4
7	[84.7, 100.0]	15.3 ± 2.4

**Table 2:** Mean time (%) of maximum normalized peaks of the drinking ADL in dominant and non-dominant arm muscles.

Arm	Muscle	Mean Time of Amplitude Peak (%)	Mean Amplitude Contraction Peak (mV)
Dominant	PM	38.3	0.3
	AD	42.0	0.9
	MD	43.1	0.4
	PD	42.6	0.2
	UT	36.1	0.6
	LT	40.7	0.4
Non-Dominant	PM	44.8	0.4
	AD	39.1	0.2
	MD	40.2	0.1
	PD	28.0	0.1
	UT	54.8	0.4
	LT	53.3	0.4

**Table 3:** Mean time (%) of maximum normalized peaks and mean joint range of motion of the drinking ADL in dominant and non-dominant arms.

Arm	Motion	Mean Time of Amplitude Peak (%)	Mean Arm Joint Range of Motion (°)
Dominant	F	100.0	−1.3
	E	70.3	8.9
	MR	100.0	−1.0
	LR	45.5	20.1
	ABD	83.0	1.4
	ADD	47.7	−28.1
Non-Dominant	F	84.7	−0.2
	E	58.5	0.1
	MR	36.2	0.5
	LR	100.0	−0.1
	ABD	100.0	0.1
	ADD	52.5	0.9

**Table 4:** Mean normalized time interval (s) and mean phase duration (%) of the eating ADL phase.

Phases	Interval of Mean Time (%)	Mean Duration ± SDT (%)
1	[0, 15.9]	15.9 ± 2.5
2	[15.9, 21.1]	5.2 ± 1.7
3	[21.1, 44.1]	23.0 ± 3.0
4	[44.1, 55.7]	11.6 ± 2.7
5	[55.7, 69.5]	13.7 ± 2.9
6	[69.5, 86.2]	16.8 ± 3.9
7	[86.2, 100.0]	13.8 ± 1.9

**Table 5:** Mean time (%) of maximum normalized peaks of the eating ADL in dominant and non-dominant arm muscles.

Arm	Muscle	Mean Time of Amplitude Peak (%)	Mean Amplitude Contraction Peak (mV)
Dominant	PM	47.8	0.3
	AD	46.3	0.7
	MD	46.7	0.4
	PD	78.0	0.3
	UT	46.1	0.8
	LT	47.9	0.4
Non-Dominant	PM	82.0	0.4
	AD	79.5	0.2
	MD	52.2	0.2
	PD	59.9	0.2
	UT	47.0	0.6
	LT	58.1	0.3

**Table 6:** Mean time (%) of maximum normalized peaks and mean joint range of motion of the eating ADL in dominant and non-dominant arms.

Arm	Motion	Mean Time of Amplitude Peak (%)	Mean Arm Joint Range of Motion (°)
Dominant	F	80.5	−3.6
	E	44.9	6.1
	MR	23.8	−2.7
	LR	49.2	6.1
	ABD	12.1	0.9
	ADD	51.7	−6.0
Non-Dominant	F	100.0	0.0
	E	55.4	0.3
	MR	31.4	1.0
	LR	100.0	−0.1
	ABD	100.0	0.2
	ADD	63.6	1.3

## Data Availability

All data reported in this study, as well as the codes developed for the analyses, are available to any researcher upon any reasonable request sent to the corresponding authors.
